# The Composition and Spatial Patterns of Bacterial Virulence Factors and Antibiotic Resistance Genes in 19 Wastewater Treatment Plants

**DOI:** 10.1371/journal.pone.0167422

**Published:** 2016-12-01

**Authors:** Bing Zhang, Yu Xia, Xianghua Wen, Xiaohui Wang, Yunfeng Yang, Jizhong Zhou, Yu Zhang

**Affiliations:** 1 Environmental Simulation and Pollution Control State Key Joint Laboratory, School of Environment, Tsinghua University, Beijing, China; 2 Institute for Environmental Genomics, Department of Microbiology and Plant Biology and School of Civil Engineering and Environmental Sciences, University of Oklahoma, Norman, Oklahoma, United States of America; 3 Earth Science Division, Lawrence Berkeley National Laboratory, Berkeley, California, United States of America; 4 State Key Laboratory of Environmental Aquatic Chemistry, Research Center for Eco-environmental Sciences, Chinese Academy of Sciences, Beijing, China; Free University of Bozen/Bolzano, ITALY

## Abstract

Bacterial pathogenicity and antibiotic resistance are of concern for environmental safety and public health. Accumulating evidence suggests that wastewater treatment plants (WWTPs) are as an important sink and source of pathogens and antibiotic resistance genes (ARGs). Virulence genes (encoding virulence factors) are good indicators for bacterial pathogenic potentials. To achieve a comprehensive understanding of bacterial pathogenic potentials and antibiotic resistance in WWTPs, bacterial virulence genes and ARGs in 19 WWTPs covering a majority of latitudinal zones of China were surveyed by using GeoChip 4.2. A total of 1610 genes covering 13 virulence factors and 1903 genes belonging to 11 ARG families were detected respectively. The bacterial virulence genes exhibited significant spatial distribution patterns of a latitudinal biodiversity gradient and a distance-decay relationship across China. Moreover, virulence genes tended to coexist with ARGs as shown by their strongly positive associations. In addition, key environmental factors shaping the overall virulence gene structure were identified. This study profiles the occurrence, composition and distribution of virulence genes and ARGs in current WWTPs in China, and uncovers spatial patterns and important environmental variables shaping their structure, which may provide the basis for further studies of bacterial virulence factors and antibiotic resistance in WWTPs.

## Introduction

Although great efforts have been made in recent years to control the distribution of bacterial pathogens in the environment, they still pose a large world-wide threat to public health and the environment [1, 2]. Many of pathogens are opportunistic and reside most of their life cycle in non-host environments [3],but they can be transmitted to hosts, including humans, and cause outbreaks and epidemics [4] in certain conditions. Essentially, the ability of bacterial pathogens to establish infection and cause disease is directly or indirectly determined by multiple virulence factors acting individually or together [5, 6]. Virulence factors as elements encoded by genes [7] can be divided into several categories on the basis of the mechanism of virulence and function [8], such as adherence, colonization, immune evasion, secretion system, invasion, toxin production and iron uptake [9]. Friman et al. [3] have reported that bacterial virulence correlated with their survival capability positively in environmental reservoirs. Furthermore, virulence genes (encoding virulence factors) are recognized to be more specific as genetic targets for the detection of bacterial pathogens than the 16S rRNA gene due to the limited ability of the 16S rRNA gene to differentiate closely related microorganisms [10]. Therefore, information on the virulence properties of an environment, such as abundance, distribution and their correlation with environmental properties, is critical to understand the nature and extent of their potential threat [5].

Another growing concern is that more and more bacterial pathogens have become resistant to antibiotics [11]. As the “gut” of a city, wastewater treatment plants (WWTPs) receive a large variety of contaminants, including antibiotics and pathogens [12]. Because of variable mixtures of bacteria, abundant nutrients and antimicrobial agents, WWTPs is considered a hotpot for the spread of antibiotic resistance genes via horizontal gene transfer [11, 13, 14]. However, WWTPs mostly using biological treatment process (activated sludge process), focus on the removal of physical and chemical pollutants while overlooking biological contaminants [15]. A wide range of pathogens and ARGs have been detected in activated sludge and effluent of WWTPs [16, 17]. Growing evidences suggest that clinical resistance is intimately associated with environmental ARGs and bacteria [18]. It is thus critical to focus on ARGs in overall microbial communities as well as those associated with virulence genes.

Moreover, centralized WWTPs are widely used in cities and represent similar habitats because of receiving similar domestic wastewater and operating under relatively similar conditions [19], but microbial communities within these reactors are highly diverse, dynamic and complex [20]. Therefore, WWTPs are ideal model systems to test whether and how the spatial distribution patterns of virulence genes in eco-systems. However, current knowledge of bacterial virulence factors and ARGs in WWTPs and their corresponding spatial patterns is incomplete.

Previous studies have been successful to assess pathogenic properties [5] and antibiotic resistance [21] of microbial community using Geochip-based array (PathoChip) or GeoChip. It also has been demonstrated that GeoChip has a good specificity, sensitivity, and quantitation [9, 22–25], which can be a reliable and comprehensive tool to investigate virulence genes and ARGs simultaneously. In addition, the probes in Geochip are designed on the basis of the gene sequences from pure cultures of known phylogeny or from environmental sequences of known taxonomic groups based on the National Center for Biotechnology Information (NCBI) database, so the hybridization data can be used to assess phylogenetic of composition and structure of microbial communities [26].

In this study, we utilized the GeoChip 4.2 to characterize bacterial virulence genes and ARGs in 19 WWTPs which cover a majority of latitudinal zones of China to address the following questions: (i) what is the spatial distribution of virulence genes? (ii) What are the key environmental factors affecting the structure of virulence genes? (iii) What is the relationship between virulence genes and ARGs?

## Materials and Methods

### Sample collection and DNA extraction

Nineteen full-scale wastewater treatment systems located in 7 different cities of China, including Dalian (DL1, DL2, DL3), Beijing (BJ1, BJ2, BJ3, BJ4) and Zhengzhou (ZZ1, ZZ2, ZZ3), Changsha (CS1, CS2), Wuxi (WX1, WX2), Shanghai (SH1, SH2) and Shenzhen (SZ1, SZ2, SZ3) were investigated in this study. A map of these cities’ geographic locations is shown in S1 Fig. Details of the ID code, locations, influent characteristics and operational parameters of the 19 WWTPs are listed in Table 1. No specific permits were required for the described field studies. We confirm that: i) the 97 locations were not privately-owned or protected in any way; and ii) the field studies did not involve endangered or protected species.

**Table 1:** Characteristics of 19 geographically distributed wastewater treatment plants (WWTPs).

WWTPs Code	Geographic Location			Environmental Variable							
	City	Longitude	latitude	IN_COD(mg/liter)	IN_TN(mg/liter)	IN_NH_4_^+^(mg/liter)	IN_pH (mg/liter)	IN_TP (mg/liter)	Temp.(℃)	DO (mg/liter)	pH
BJ1	Beijing	116.30	40.02	150.67	48.70	42.46	7.25	6.10	19.80	2.94	7.10
BJ2	Beijing	116.44	39.83	443.00	46.00	36.00	7.38	6.80	26.00	1.10	6.91
BJ3	Beijing	116.36	40.03	437.10	64.73	58.61	7.23	6.12	23.10	3.75	7.15
BJ4	Beijing	116.53	39.91	452.80	58.00	52.47	6.97	6.00	20.70	1.39	6.97
CS1	Changsha	113.06	28.22	177.14	20.00	20.00	7.14	2.00	25.10	4.60	6.81
CS2	Changsha	113.02	28.18	74.67	25.00	25.00	7.26	1.80	25.00	3.20	6.98
DL1	Dalian	121.50	38.86	243.00	28.00	37.49	7.37	5.35	15.30	5.53	6.72
DL2	Dalian	121.64	39.00	258.00	24.00	39.35	7.43	5.86	14.10	7.00	6.81
DL3	Dalian	121.68	38.88	291.00	18.19	16.40	7.31	5.81	13.70	7.46	6.51
SH1	Shanghai	121.62	31.34	281.94	58.00	41.00	7.30	6.00	26.80	2.80	7.82
SH2	Shanghai	121.49	31.28	300.00	64.00	38.70	7.55	7.20	26.40	2.80	7.53
SZ1	Shenzhen	114.15	22.54	317.00	33.00	27.00	6.91	4.30	25.00	4.49	6.42
SZ2	Shenzhen	114.15	22.54	328.00	33.00	26.00	6.91	4.30	24.60	2.99	6.84
SZ3	Shenzhen	114.10	22.53	343.00	77.90	55.00	7.13	5.10	30.70	2.60	6.41
WX1	Wuxi	120.32	31.53	374.89	45.58	45.35	7.24	7.24	18.40	2.35	6.88
WX2	Wuxi	120.32	31.53	374.89	45.58	45.35	7.24	7.24	18.05	1.54	6.72
ZZ1	Zhengzhou	113.74	34.85	427.00	50.60	37.40	7.87	10.80	17.00	3.00	6.80
ZZ2	Zhengzhou	113.80	34.78	443.73	55.72	47.79	7.76	5.73	17.00	3.00	6.90
ZZ3	Zhengzhou	113.80	34.78	443.73	55.72	47.79	7.76	5.73	17.00	3.00	6.90

The alphabets (DL, BJ, ZZ, CS, WX, SH, SZ) represent the cities and the number represents the number of the WWTPs. Abbreviation: IN_COD, chemical oxygen demand (COD) of influent; IN_TN, total nitrogen (TN) of influent, IN_NH_4_^+^, ammonia of influent; IN_pH, pH of influent; IN_TP, total phosphorus of influent; temp., temperature of activated sludge; DO, dissolved oxygen (DO) in activated sludge; pH, pH of activated sludge.

In each WWTP, triplicate activated sludge samples were collected from the end part of the aeration tank once a day for three consecutive days in the summer of 2011. Firstly, the samples were collected into vessel and briefly settled on site to be concentrated. Then, they were fixed in 50% (v/v) ethanol aqueous solution. The fixed samples were transported to laboratory on ice. Each 30-ml sample was dispensed into a 50-ml sterile tube and centrifuged at 15,000g for 10 min. The supernatant was decanted and the pellets were stored at -80°C until DNA was extracted. Meanwhile, the pollutant concentrations of influents and effluents were measured using standard methods, including ammonia, total nitrogen, total phosphorus and chemical oxygen demand (COD). Temperature, pH and dissolved oxygen (DO) were also measured *in situ*. DNA was extracted from the pellets of activated sludge samples by joint use of freezing and sodium dodecyl sulfate (SDS) for cell lysis [27]. Wizard® SV Genomic DNA Purification Kit was then employed to pure the extracted products (Promega, Madison, WI). After purification, DNA quality was assayed by using ND-2000 spectrophotometer (Nanodrop Inc., Wilmington, DE, USA). To ensure sample quality, the ratio of absorbance at 260 and 280 nm was required to be between 1.80 and 2.0, and the ratio of absorbance at 260 and 230 nm needed to be higher than 1.70.

### DNA labeling, and hybridization

DNA (1μg) was labeled, purified and dried before hybridization using previously described methods [28]. All labeled DNA was suspended again in a 10-μl hybridization solution as previously described [29]. Subsequently, DNA was hybridized at 42°C with 40% formamide for 16 hours on a MAUI hybridization station (BioMicro, Salt Lake City, UT, USA) utilizing GeoChip 4.2. In essence, GeoChip 4.2 is a functional gene microarray containing 83,992 oligonucleotide (50-mer) probes (nucleic acid sequence) from the genes with known biological functions [25] and can cover as much as 152,414 coding sequencing in 410 gene categories. Specifically, GeoChip 4.2 contained a total of 3729 probes belonging to 13 bacterial virulence gene families (the virulence genes encoding the same type of virulence factors are classified as a gene family) present in most bacterial pathogens and 3334 probes targeting 11 ARG families (the ARGs with the same function are classified as one ARG family) [30, 31]. Briefly, 13 virulence gene families were included which encode protein related to adhesion, aerobactin, capsule colonization factors, fimbriae, hemolysin, invasion, iron oxidation, pilin, secretion, sortase, toxin and virulence protein [9] (description of each virulence factor can be found in S1 Table). Five transporters (ATP-binding cassette (ABC), multidrug and toxic compound exporters (MATE), the resistance-nodulation-division (RND), major facilitator superfamily (MFS) and small multidrug resistance (SMR)), four classes of beta-lactam resistance (beta-lactamase class A to D), tetracycline resistance and vancomycin resistance were selected to analyzed bacterial antibiotic resistance (S2 Table). Detailed information about individual probe can be downloaded at www.ou.edu/ieg/tools.html. Microarrays were scanned by a Nimblegen (Madison, WI) MS200 Microarray scanner at 100% laser power.

### Data analysis

Spots with a signal-to-noise ration of <2 and outliers of replicates were removed and signal intensities were normalized with and across samples based on the mean signal intensity as described previously [24]. The normalized hybridization data for virulence genes sequences and antibiotic resistance genes sequences were selected based on gene families and functional groups.

Shannon-Winner index (H), evenness index (E), Canonical correspondence analysis (CCA), analysis of variance (ANOVA) and partial Mantel test of 57 samples were determined using R (v.2.13.1; http://www.r-project.org/). Spatial variables measured as latitude-longitude coordinates were converted into projected coordinates and are represented by a cubic trend-surface polynomial to capture broad-scale spatial trends [32]. Distance-decay analyses were constructed using R based on Jaccrad distance and Bray-Curtis indices. Z values, as a measure of the rate of bacterial virulence genes richness increasing with distance were calculated as the slope of a linear least squares regression on the relationship between geographic distances (log-transformed) versus bacterial virulence genes similarity (log-transformed).

### Data accession number

Detailed information of the detected genes and their signal intensities is available at the Gene Expression Omnibus (www.ncbi.nlm.nih.gov/geo/, accession number GSE54055).

## Results and Discussion

### Overall virulence gene structure and composition

**Fig 1:**
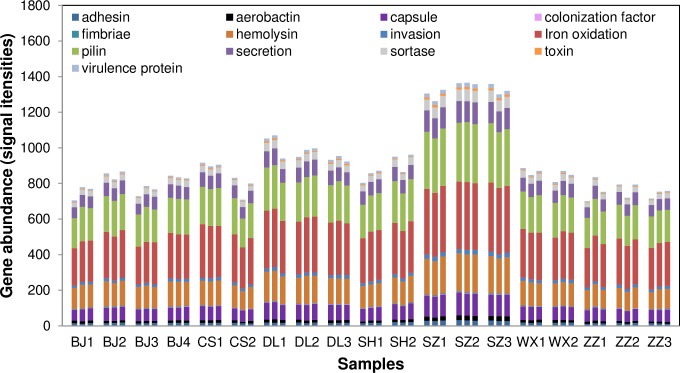
Gene abundance of each virulence gene family in 57 samples. For clear and pithy description, we use the names of virulence factors to substitute the related names of virulence genes in Fig 1.

A total of 1610 genes (probes), covering 13 categories (families) of bacterial virulence factors, were detected (S3 Table). Among these, 1438 genes (89.3%) came from *Proteobacteria*. The virulence genes of activated sludge communities detected in this study showed had higher richness than those of soil (1372 genes detected) and deep-sea water (372 genes) communities [5]. In each WWTP, the 13 virulence gene families showed similar abundance distribution patterns, but they were of different abundances (Fig 1). Genes encoding toxin were more abundant in SZ1, SZ2 and SZ3. About each gene family, genes encoding siderophore were dominant both in number (26.8%-33.4%) (numbers of detected genes encoding each individual virulence factor in 57 samples are shown in S3 Table) and in abundance (27.6%-33.7%) (S2 Fig). Siderophores are small, high-affinity iron-chelating compounds, which play roles in scavenging iron from iron-limiting environment and making the mineral available to the microbial cell [33]. Though siderophores as a nonspecific virulence factor are indirectly involved in pathogenesis, they are as equally important as specific ones for bacteria to establish infection [7]. Our results showed that virulence genes encoding siderophores came from many hydrocarbon-degrading bacteria, such as *Delftia acidovorans* SPH-1 (GenBank: CP000884.1), *Novosphinggobium aromaticivorans* DSM 1244 (GenBank: CP000248.1) and *Variovorax paradoxus* S110 (GenBank: CP001635.1), which are consistent with the study about virulence genes in oil-contaminated seawater [5]. The virulence genes encoding siderophores may facilitate the uptake of iron [5] and increase the potential of pollutants degradation in WWTPs.

To assess the virulence gene diversity in each sample, richness, evenness and Shannon-Weaver index (H) were calculated. Seven hundred and two (BJ1A) to 1370 (SZ2B) genes were detected within each sample (S3 Table). The values of H ranged from 6.55 to 7.22 across the 19 WWTPs. Both the highest abundance and greatest diversity of virulence genes came from the SZ, indicating a possible increase in abundance and diversity of pathogenic bacteria under environmental perturbations such as high temperature [5]. The evenness of all samples did not show significant difference (S4 Table).

### Relationships between virulence genes and environmental factors

**Fig 2:**
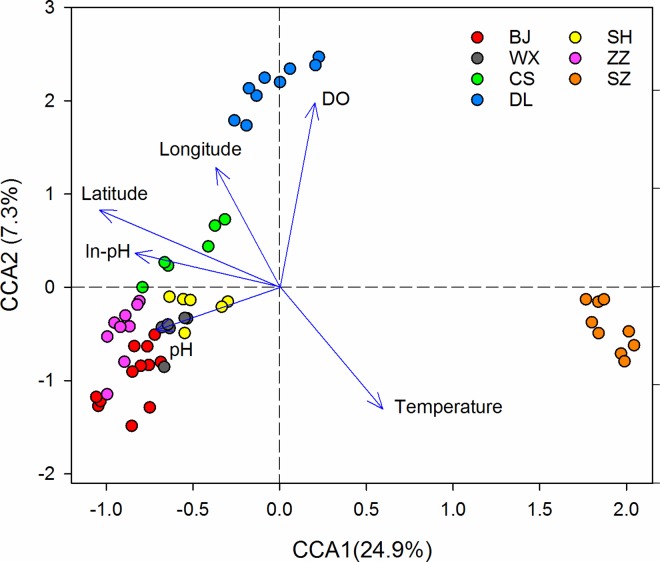
Canonical correspondence analysis (CCA) of 57 samples based on the bacterial virulence genes. Arrows indicate the direction and magnitude of measurable variables associated with community structures. The solid circles represent the different WWTPs. In-pH represents the pH of influent.

Understanding the factors that shape the bacterial virulence genes structure in WWTPs could potentially enhance treatment performance and control [34]. In this study, the effects of the 10 measured factors (Table 1) were evaluated including geographic location of the WWTPs and environmental factors which are usually monitored in routine measurement of WWTPs. CCA was performed to identify the key environmental attributes shaping the virulence gene structure from these 10 factors. Based on values of variance inflation factors (VIF) less than 20 with 999 Monte Carlo permutations, six environmental variables, latitude, longitude, pH of influent, dissolve oxygen (DO), temperature and pH of activated sludge were selected in the CCA biplot (Fig 2). Among them, latitude and pH showed a strong negative correlation with the first axis and DO showed a strong positive correlation with the second axis. These six factors explained 43.41% virulence gene structure in total and fitted CCA model very well as a whole (*P*<0.001). There was also a large proportion of variance that could not be explained, indicating that unmeasured biotic and abiotic environmental factors may also play important roles in shaping virulence gene structure.

In general, bacterial virulence genes from the same city were clustered together and communities in different WWTPs were separated based on latitude, pH and temperature along the first axis. Virulence gene structure in WWTPs DL1, DL2 and DL3 were primarily linked to DO, which would explain why virulence genes in DL were separated from the others.

As shown in Fig 2, latitude had a strong effect on the overall virulence genes structure, which was confirmed by the partial Mantel test. The partial Mantel test result showed that overall virulence gene composition was strongly correlated to latitude (r = 0.55, *P*<0.01).This was consistent with previous observations that latitude could best explain community variations of influent sewage samples from 13 different WWTPs [35]. The possible reasons are that the structure of virulence genes is a reflection of the local epidemic situation to some degree and the local ambient environment and climate would influence both source microorganisms entering the system and resident microorganisms in the WWTPs [36].

Temperature also has a strong effect on virulence gene structure. In addition, there were positive correlations between temperature and abundance of virulence genes (r = 0.38, P<0.01) and between temperature and diversity (r = 0.36, P<0.01), respectively, which mean that elevated temperature can lead to an increase in the relative abundance and diversity of virulence genes [5]. Konkel et al [37] pointed that virulence genes expression can be regulated by temperature, which acts as an “on-off” switch in a manner distinct from the more general heat-shock response. As such, these findings may indicate that temperature is vital environmental variable and provide the basis for the development of method to control virulence factors in activated sludge.

### Spatial distribution patterns

**Fig 3:**
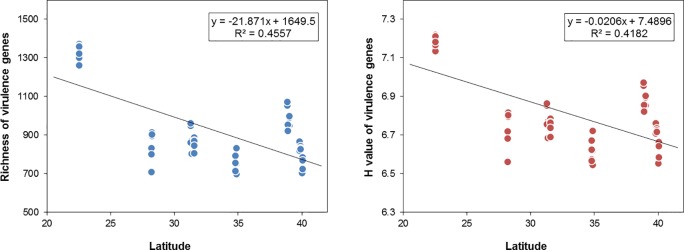
A. Latitudinal gradient of richness. B. Latitudinal gradient of H value (Shannon-Wiener index).

As we mentioned previously, WWTPs are ideal model systems to test whether and how the spatial distribution patterns of virulence genes in eco-systems. Firstly, the pattern of a latitudinal biodiversity gradient was evaluated, that is, whether species richness or diversity increases towards lower latitudes [38, 39]. The latitudinal gradient of alpha diversity was analyzed by a linear regression. Strong correlations between richness and latitude (*r* = 0.69, *P*<0.01) as well as H value and latitude (*r* = 0.66, *P*<0.01), were detected, respectively (Fig 3), suggesting that virulence genes in WWTPs had a latitudinal diversity gradient.

**Fig 4:**
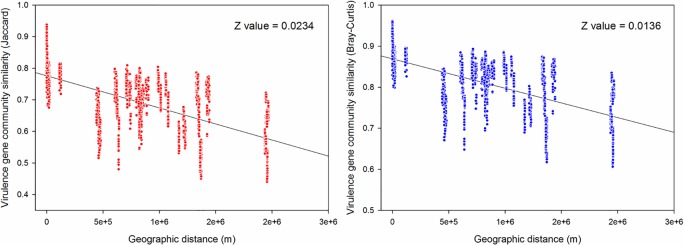
**A. Distance-decay patterns for virulence genes in activated sludge on Bray-Curtis similarity index. B. Distance-decay patterns for virulence genes in activated sludge on Jaccard similarity index.** Each dot represents a pairwise similarity of virulence genes. S means virulence gene similarity and D means geographic distance.

The bacterial virulence genes as whole displayed a significant, negative distance-decay relationship based on Bray-Cutis similarity (r = -0.49, P<0.01(Fig 4A) and Jaccard similarity (r = -0.51, P<0.01) (Fig 4B), indicating that the composition of bacterial virulence genes in WWTPs located proximally were more similar than that in WWTPs located father apart. The z value based on Bray-Cutis distance for the whole virulence genes was 0.0136, while z value based on Jaccard distance was 0.0234 (higher than the former). Since the Bray-Cutis index focuses on the difference in species abundance between two communities and the Jaccard index is the ratio of the number of shared species to the number of whole distinct species in two samples, our finding suggest that dominant virulence genes had a lower turnover in WWTPs. Furthermore, the z values obtained in this study are several times lower than those for animals and plants, which was 0.27 and 0.32 respectively [19]. In addition, they were lower than those observed in other habitats for microorganisms. For example, the z values for metal resistance genes families in a forest soil were 0.0616 and z values varied considerably across different functional and phylogenetic groups (z = 0.0475–0.0959) [40]. It is because similar functions and relatively similar operational conditions in WWTPs may result in lower turnovers[19].

### Distribution of ARGs in WWTPs

There were 3334 ARGs (589 sequence-specific genes and 2745 group-specific genes) covering 5533 sequence in GeoChip 4.2. A total of 1903 ARGs deriving from 807 species (strains) were detected in this study. Many of species (strains) are related to degradation of pollutants, such as *Agrobacterium radiobacter K84* (GenBank: NC_011985.1), *Nitrosomonas sp*. *AL212* (GenBank: CP002552.1), which demonstrates that various microorganisms had the capability of antibiotic resistance and these bacteria can act as potential reservoirs of resistance genes[41]. SampleSZ2A showed the highest richness (1419 ARGs), whereas sample ZZ1A had the lowest richness (677 ARGs).

**Fig 5:**
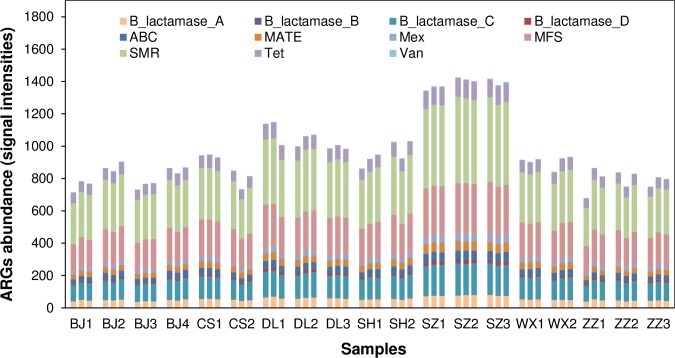
Abundance of total detected ARGs in 57 WWTPs samples. Abbreviations: B_lactamase_A, B, C, D, genes encoding beta-lactamase class A, B, C, D, respectively; ABC, genes encoding ATP-binding cassette transporter; MATE, genes encoding multidrug and toxic compound exporters; NRD, genes encoding the resistance-nodulation-division transporter; MFS, genes encoding major facilitator superfamily transporter; SMR, genes encoding small multidrug resistance; Tet, genes encoding tetracycline resistance protein; Van, genes encoding vancomycin resistance protein.

All these detected ARGs belong to 11 antibiotic resistance categories, and ten of them were shared by all 57 WWTPs samples (Fig 5), which indicated that antibiotic resistance are prevalent and widely disseminated in non-clinical environments[42]. ARGs belonging to ABC, MATE, MFS and SMR were widely distributed in both Gram-positive and negative bacteria, while genes encoding RND only derived from Gram-negative bacteria, which agreed with the study from Handzlik et al. [43]. For each gene family, SMR transporters had the highest abundance, which were also found as the most dominant ARGs in pharmaceutical wastewater treatment systems [21]. All five transporters accounted for 70%±0.12% (abundance) of all detected ARGs, indicating that efflux pumps are the major antibiotic resistance determines [14]. One concern about array-based technology is that all of the probes on the array are derived from a chosen set of genes and sequences [22] and we need to admit that some genes like aminoglycoside -resistance genes were lack in order to ensure the array specificity. However, it is not easy for microarrays to cover all of the known genes. As we know, sequences of a particular functional gene are highly homologous and/or incomplete, especially sequences derived from laboratory cloning of environmental samples [25]. In order to meet the critical criteria for designing probes and ensure the array specificity, lots of sequences need to be discarded. But, the designers are always keeping increasing the coverage of GeoChip based on the latest database. The coverage of virulence genes and antibiotic resistance genes on GeoChip 4.2 was manually checked and improved by modifying keyword queries for some genes and increasing gene sequence coverage from the latest public database at the time of development [9]. Therefore, GeoChip 4.2, as high-throughput technology, can cover as many as 3729 genes related to 13 major virulence factors and 3334 genes related to 4 categories of antibiotic resistance, which can be reliable and comprehensive for analyzing bacterial virulence and antibiotic resistance simultaneously and avoid the limitation of primer selection compared to PCR.

Bacteria can intrinsically resist to particular antibiotics but can also acquire or develop resistance to antibiotics [44]. Intrinsic resistance is a natural characteristic of a bacterial species by reducing uptake or inactivating antibiotics [44]. For example, *E*. *coli* has innate resistance to vancomycin [45]. Also, Gram-negative bacteria are intrinsically resistant to many antibiotics with the help of efflux pumps [44]. On the contrary, acquired resistance may arise by mutation in chromosomal genes or by the acquisition of mobile genetic elements (MGEs), e.g. plasmids, transposons and integrons [46, 47]. For instance, *tet* genes which also had high abundance in activated sludge samples, are often located on plasmids and can be horizontally transferred among bacterial strains in wastewater treatment plants [48].

### Correlation between virulence genes and ARGs

It has been evidenced that acquisition of antibiotic resistance determines via horizontal gene transfer (HGT) play an important role in the development and spread of antibiotic resistance among pathogenic bacteria [49, 50]. Since activated sludge with high microbial density and diversity can facilitate HGT of ARGs among bacteria [34, 40, 41], pathogenic bacteria in activated sludge have large potential to develop antibiotic resistance. However, the relationship between virulence genes and ARGs has rarely been studied. The mantel test results indicated that the virulence genes as a whole were positively correlated with the ARGs as a whole (r = 0.98, P<0.01).

One of the plausible explanations for why a positive association between virulence genes and ARGs was observed is that opportunistic pathogens tended to harbor ARGs. Some previous studies showed that long-term exposure to sub-inhibitory concentrations of antibiotics in wastewater may favor ARGs horizontal transfer in activated sludge [51]and also could favor bacterial virulence under certain conditions [46]. These opportunistic pathogens with resistance to antibiotics could pose potential threat to public health when released into the receiving surface water.

In addition, small multidrug resistance efflux transporters (SMR), with the highest abundance in activated sludge, are involved in other processes such as virulence [46], and multidrug efflux systems can directly export virulent determinants and contribute to bacterial pathogenesis [52].

## Conclusion

In sum, the study not only provided insight into the structure and diversity of bacterial virulence genes in geographically distributed WWTPs, but also revealed the correlation of the structures of virulence genes and ARGs. Overall, 1610 virulence genes covering all 13 bacterial virulence factors and 1903 ARGs belonging to 11 ARG families were detected in 57 WWTPs samples. Virulence genes in WWTPs showed a clear latitudinal biodiversity gradient and distance-decay relationship across China. Moreover, abundances of virulence genes and ARGs showed a strong and positive correlation. These results provide us with more comprehensive information on virulence genes and ARGs in WWTPs, which may facilitate a more targeted control and reduce potential risks in the future.

## Supporting Information

S1 Fig The geographic locations of sampling cities. (TIF)

S2 Fig Relative gene abundance of 13 virulence gene families in 57 samples. For clear and pithy description, we use the names of virulence factors to substitute the related names of virulence genes in S2 Fig.
(TIF)

S1 Table The details of virulence gene families in GeoChip 4.2. (DOCX)

S2 Table The details of antibiotic resistance gene families in GeoChip 4.2. (DOCX)

S3 Table Distribution of detected virulence gene families in WWTPs. The alphabets (DL, BJ, ZZ, CS, WX, SH, SZ) represent the cities and the number represents the number of the WWTPs. ‘A’, “B”, “C” following number mean three replicates in the same WWTP respectively.
(DOCX)

S4 Table Diversity indices from 57 samples. The alphabets (DL, BJ, ZZ, CS, WX, SH, SZ) represent the cities and the number represents the number of the WWTPs. ‘A’, “B”, “C” following number mean three replicates in the same WWTP respectively.
(DOCX)
