# Supplementary figures and images for: The Composition and Spatial Patterns of Bacterial Virulence Factors and Antibiotic Resistance Genes in 19 Wastewater Treatment Plants
Source: PLoS One. 2016 Dec 1;11(12):e0167422. doi: 10.1371/journal.pone.0167422 (PMC5132249; doi:10.1371/journal.pone.0167422)

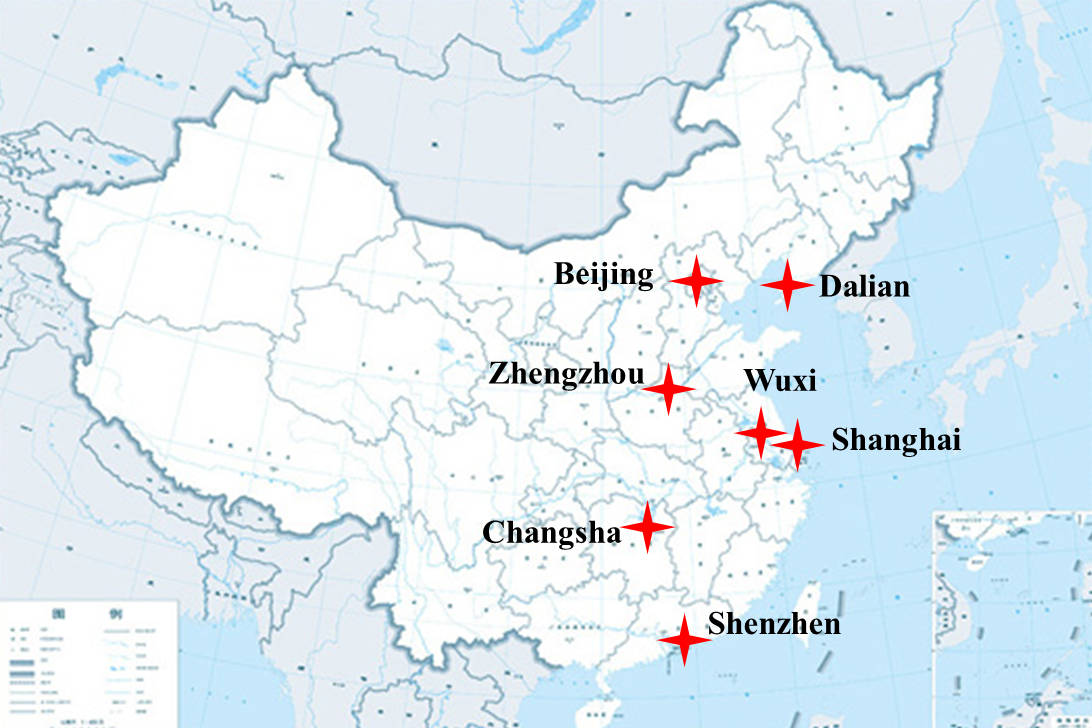

Supplement: S1 Fig — (TIF) [file pone.0167422.s001.tif]

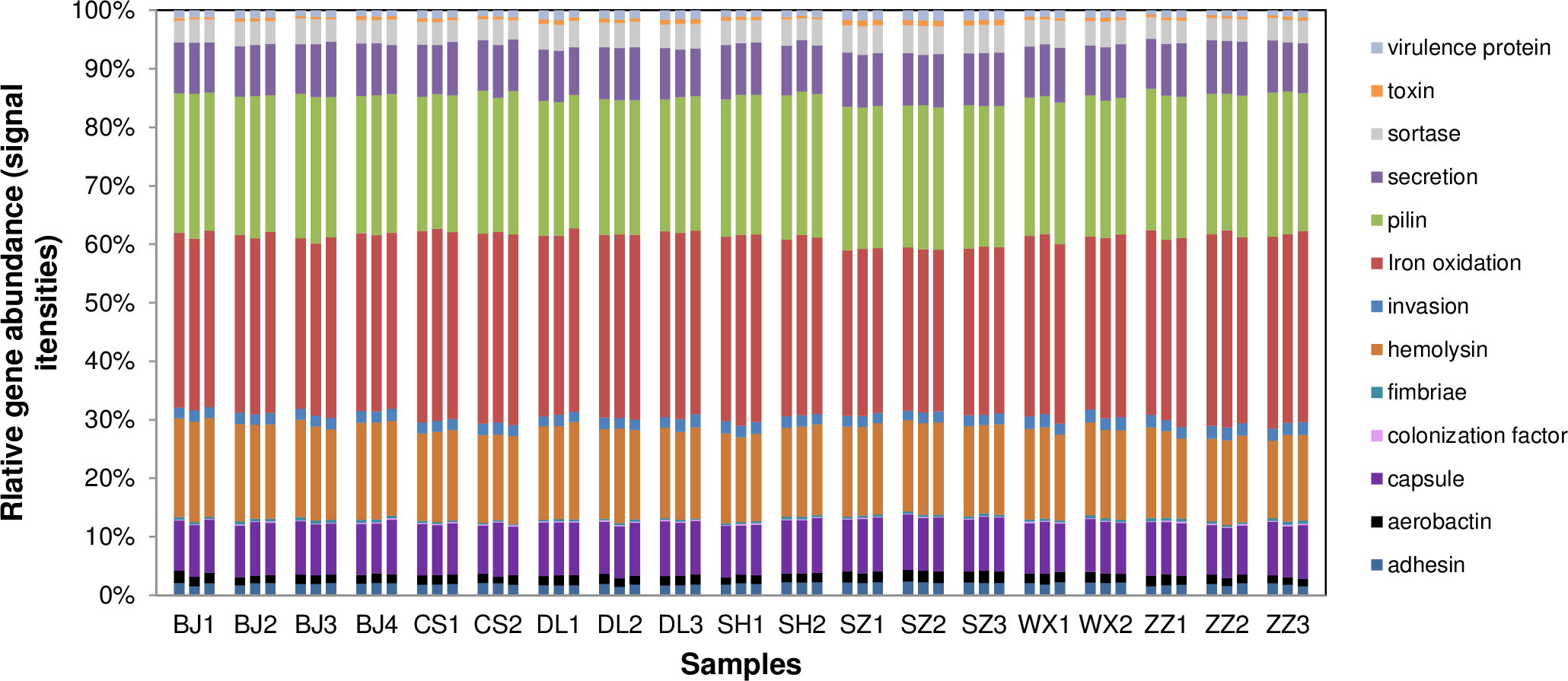

Supplement: S2 Fig — For clear and pithy description, we use the names of virulence factors to substitute the related names of virulence genes in S2 Fig. (TIF) [file pone.0167422.s002.tif]
