# Supplementary material for: The Composition and Spatial Patterns of Bacterial Virulence Factors and Antibiotic Resistance Genes in 19 Wastewater Treatment Plants
Source: PLoS One. 2016 Dec 1;11(12):e0167422. doi: 10.1371/journal.pone.0167422 (PMC5132249; doi:10.1371/journal.pone.0167422)
Supplement: S1 Table — (DOCX) [file pone.0167422.s003.docx]

**S1 Table. The details of virulence gene families in GeoChip 4.2**

| virulence gene family names in GeoChip4.2 | Enzyme/protein name | Probes contained | Annotation |
| --- | --- | --- | --- |
| pap | Adhesion | 113 | cell-surface components or appendages of bacteria to promote bacterial colonization within their hosts[1] |
| inv | Invasion | 65 | Proteins that either disrupt host cell membranes or stimulate their own endocytosis or macro-pinocytosis into host cells. These virulence factors allow the bacteria to enter host cells and facilitate entry into the body across epithelial tissue layers at the body surface. |
| colonization | Colonization factor | 26 | Surface structures that allow bacteria to bind and colonize onto host cells [2] |
| iuc | Aerobactin | 126 | a bacterial iron chelating agent |
| type_III_secretion | Secretion | 288 | Particular prevalent among Gram-negative bacterial pathogens to inject effectors directly into their hosts [3] |
| cap | Capsule | 388 | promote virulence by reducing host immune responses [4] |
| fimbriae | Fimbriae | 16 | short proteinaceous appendages present in many Gram-negative bacteria and some Gram-positive bacteria and mediate the surface attachment [5] |
| hly | Hemolysin | 620 | have an important role in toxigenesis by affecting and damaging a host cell directly and aggressively[6, 7] |
| iro | Siderophore | 611 | small, high-affinity iron-chelating compounds generally produced under iron-limiting conditions to scavenge iron[8] |
| pilin | Pilin | 1038 | Major subunit protein of pili in many bacteria and play roles in surface attachment and DNA transfer by conjugation[9-11] |
| srt | Sortase | 295 | a family of enzymes, which are required for cell wall anchoring of surface proteins, adhesion to host cells and colonization of tissues[12, 13] |
| toxin | Toxin | 70 | have an important role in toxigenesis by affecting and damaging a host cell directly and aggressively[6, 7] |
| vip | Virulence protein | 73 | Including surface-exposed virulence protein BigA, iron-regulated outer membrane virulence protein IrgA, adherence and virulence protein A and virulence proteins S and Q. |

**Reference**

1. Kline KA, Falker S, Dahlberg S, Normark S, Henriques-Normark B. Bacterial Adhesins in Host-Microbe Interactions. Cell Host Microbe. 2009;5(6):580-92. doi: 10.1016/j.chom.2009.05.011. PubMed PMID: WOS:000267250900010.

2. Tobias J, Svennerholm AM. Strategies to overexpress enterotoxigenic Escherichia coli (ETEC) colonization factors for the construction of oral whole-cell inactivated ETEC vaccine candidates. Applied microbiology and biotechnology. 2012;93(6):2291-300. doi: 10.1007/s00253-012-3930-6. PubMed PMID: WOS:000301181600004.

3. Galan JE, Collmer A. Type III secretion machines: Bacterial devices for protein delivery into host cells. Science. 1999;284(5418):1322-8. doi: DOI 10.1126/science.284.5418.1322. PubMed PMID: WOS:000080430600044.

4. Singh A, Wyant T, Anaya-Bergman C, Aduse-Opoku J, Brunner J, Laine ML, et al. The Capsule of Porphyromonas gingivalis Leads to a Reduction in the Host Inflammatory Response, Evasion of Phagocytosis, and Increase in Virulence. Infect Immun. 2011;79(11):4533-42. doi: 10.1128/Iai.05016-11. PubMed PMID: WOS:000296352400024.

5. Blomfield IC, Calie PJ, Eberhardt KJ, Mcclain MS, Eisenstein BI. Lrp Stimulates Phase Variation of Type-1 Fimbriation in Escherichia-Coli K-12. Journal of Bacteriology. 1993;175(1):27-36. PubMed PMID: WOS:A1993KE61800005.

6. Lee YJ, van Nostrand JD, Tu QC, Lu ZM, Cheng L, Yuan T, et al. The PathoChip, a functional gene array for assessing pathogenic properties of diverse microbial communities. Isme Journal. 2013;7(10):1974-84. doi: 10.1038/ismej.2013.88. PubMed PMID: WOS:000324869400009.

7. Tu Q, Yu H, He Z, Deng Y, Wu L, Van Nostrand JD, et al. GeoChip 4: a functional gene-array-based high-throughput environmental technology for microbial community analysis. Mol Ecol Resour. 2014;14(5):914-28. doi: 10.1111/1755-0998.12239. PubMed PMID: 24520909.

8. Neilands JB. Siderophores - Structure and Function of Microbial Iron Transport Compounds. J Biol Chem. 1995;270(45):26723-6. PubMed PMID: WOS:A1995TE58300001.

9. Craig L, Taylor RK, Pique ME, Adair BD, Arvai AS, Singh M, et al. Type IV pilin structure and assembly: X-ray and EM analyses of Vibrio cholerae toxin-coregulated pilus and Pseudomonas aeruginosa PAK pilin. Mol Cell. 2003;11(5):1139-50. doi: Doi 10.1016/S1097-2765(03)00170-9. PubMed PMID: WOS:000183139400006.

10. Carter MQ, Chen JS, Lory S. The Pseudomonas aeruginosa Pathogenicity Island PAPI-1 Is Transferred via a Novel Type IV Pilus. Journal of Bacteriology. 2010;192(13):3249-58. doi: 10.1128/Jb.00041-10. PubMed PMID: WOS:000278806100003.

11. Yang S, Bourne PE. The Evolutionary History of Protein Domains Viewed by Species Phylogeny. Plos One. 2009;4(12). doi: ARTN e8378

10.1371/journal.pone.0008378. PubMed PMID: WOS:000272940100014.

12. Mazmanian SK, Hung IT, Schneewind O. Sortase-catalysed anchoring of surface proteins to the cell wall of Staphylococcus aureus. Mol Microbiol. 2001;40(5):1049-57. doi: DOI 10.1046/j.1365-2958.2001.02411.x. PubMed PMID: WOS:000169579900001.

13. Cossart P, Jonquieres R. Sortase, a universal target for therapeutic agents against Gram-positive bacteria? P Natl Acad Sci USA. 2000;97(10):5013-5. doi: DOI 10.1073/pnas.97.10.5013. PubMed PMID: WOS:000086998500001.
