# Supplementary material for: The Composition and Spatial Patterns of Bacterial Virulence Factors and Antibiotic Resistance Genes in 19 Wastewater Treatment Plants
Source: PLoS One. 2016 Dec 1;11(12):e0167422. doi: 10.1371/journal.pone.0167422 (PMC5132249; doi:10.1371/journal.pone.0167422)
Supplement: S2 Table — (DOCX) [file pone.0167422.s004.docx]

**S2 Table.** **The details of antibiotic resistance gene families in GeoChip 4.2**

| Antibiotic resistance gene family names in GeoChip4.2 | Enzyme/protein name | Probes contained | Resistance type |
| --- | --- | --- | --- |
| ABC | ATP-binding cassette | 148 | Transporter |
| MATE | multidrug and toxic compound exporters | 206 | Transporter |
| NRD | the resistance-nodulation-division | 112 | Transporter |
| MFS | major facilitator superfamily | 681 | Transporter |
| SMR | small multidrug resistance | 1169 | Transporter |
| B_lactamase_A | Beta lactamases A | 185 | Beta-lactam resistance |
| B_lactamase_B | Beta lactamases B | 10 | Beta-lactam resistance |
| B_lactamase_C | Beta lactamases C | 410 | Beta-lactam resistance |
| B_lactamase_D | Beta lactamases D | 60 | Beta-lactam resistance |
| Van | Vancomycin resistance protein | 29 | Vancomycin resistance |
| Tet | Tetracycline resistance protein | 324 | Tetracycline resistance |
