# Supplementary material for: The Composition and Spatial Patterns of Bacterial Virulence Factors and Antibiotic Resistance Genes in 19 Wastewater Treatment Plants
Source: PLoS One. 2016 Dec 1;11(12):e0167422. doi: 10.1371/journal.pone.0167422 (PMC5132249; doi:10.1371/journal.pone.0167422)
Supplement: S3 Table — The alphabets (DL, BJ, ZZ, CS, WX, SH, SZ) represent the cities and the number represents the number of the WWTPs. ‘A’, “B”, “C” following number mean three replicates in the same WWTP respectively. (DOCX) [file pone.0167422.s005.docx]

**S3 Table. Distribution of detected virulence gene families in WWTPs**

| No. of probes | Virulence factors | | | | | | | | | | | | | |
| --- | --- | --- | --- | --- | --- | --- | --- | --- | --- | --- | --- | --- | --- | --- |
|  | Adhesin | Aerobactin | Capsule | Colonization factor | Fimbriae | Hemolysin | Invasion | Siderophore | Pilin | Secretion | Sortase | Toxin | Virulence protein | Grand total |
| Total probes on GeoChip | 113 | 126 | 388 | 26 | 16 | 620 | 65 | 611 | 1038 | 288 | 295 | 70 | 73 | 3729 |
| Genes in 19 WWTPs | 38 | 35 | 150 | 2 | 6 | 263 | 30 | 406 | 414 | 142 | 79 | 17 | 28 | 1610 |
| BJ1A | 15 | 15 | 60 | 1 | 3 | 119 | 13 | 208 | 169 | 61 | 26 | 3 | 9 | 702 |
| BJ1B | 12 | 13 | 68 | 1 | 3 | 134 | 15 | 226 | 194 | 67 | 31 | 3 | 9 | 776 |
| BJ1C | 16 | 14 | 69 | 1 | 3 | 130 | 14 | 230 | 182 | 65 | 30 | 3 | 9 | 766 |
| BJ2A | 15 | 12 | 75 | 2 | 4 | 143 | 17 | 257 | 202 | 74 | 36 | 5 | 12 | 854 |
| BJ2B | 17 | 11 | 75 | 1 | 4 | 132 | 14 | 244 | 201 | 71 | 33 | 5 | 11 | 819 |
| BJ2C | 19 | 12 | 77 | 2 | 4 | 140 | 17 | 264 | 204 | 76 | 34 | 5 | 11 | 865 |
| BJ3A | 14 | 12 | 66 | 1 | 4 | 121 | 14 | 209 | 180 | 61 | 32 | 3 | 7 | 724 |
| BJ3B | 15 | 12 | 68 | 1 | 4 | 126 | 15 | 229 | 197 | 71 | 34 | 3 | 9 | 784 |
| BJ3C | 17 | 11 | 67 | 1 | 4 | 120 | 15 | 233 | 186 | 72 | 29 | 3 | 9 | 767 |
| BJ4A | 17 | 13 | 73 | 2 | 4 | 141 | 17 | 253 | 199 | 75 | 34 | 6 | 8 | 842 |
| BJ4B | 18 | 14 | 70 | 2 | 4 | 139 | 16 | 248 | 201 | 74 | 33 | 5 | 9 | 833 |
| BJ4C | 17 | 13 | 77 | 2 | 4 | 135 | 17 | 246 | 198 | 69 | 36 | 5 | 8 | 827 |
| CS1A | 17 | 15 | 80 | 2 | 3 | 139 | 17 | 294 | 212 | 81 | 36 | 6 | 12 | 914 |
| CS1B | 17 | 15 | 76 | 2 | 3 | 140 | 17 | 289 | 209 | 74 | 35 | 6 | 12 | 895 |
| CS1C | 18 | 15 | 79 | 2 | 2 | 142 | 17 | 283 | 215 | 81 | 34 | 4 | 11 | 903 |
| CS2A | 18 | 14 | 68 | 2 | 2 | 126 | 16 | 266 | 205 | 71 | 30 | 4 | 8 | 830 |
| CS2B | 15 | 8 | 66 | 1 | 2 | 104 | 15 | 228 | 164 | 63 | 31 | 3 | 8 | 708 |
| CS2C | 15 | 14 | 66 | 2 | 2 | 121 | 15 | 257 | 198 | 70 | 26 | 4 | 9 | 799 |
| DL1A | 19 | 17 | 97 | 2 | 3 | 171 | 18 | 318 | 244 | 91 | 47 | 9 | 16 | 1052 |
| DL1B | 18 | 20 | 97 | 2 | 4 | 173 | 20 | 321 | 246 | 93 | 48 | 10 | 18 | 1070 |
| DL1C | 17 | 17 | 84 | 2 | 3 | 158 | 17 | 290 | 215 | 75 | 43 | 6 | 11 | 938 |
| DL2A | 19 | 17 | 84 | 2 | 3 | 146 | 19 | 291 | 223 | 83 | 41 | 7 | 13 | 948 |
| DL2B | 15 | 15 | 88 | 2 | 3 | 162 | 18 | 304 | 230 | 87 | 43 | 6 | 16 | 989 |
| DL2C | 19 | 16 | 90 | 2 | 4 | 155 | 18 | 308 | 233 | 89 | 44 | 6 | 14 | 998 |
| DL3A | 16 | 16 | 87 | 2 | 3 | 146 | 17 | 291 | 212 | 80 | 38 | 8 | 15 | 931 |
| DL3B | 17 | 16 | 86 | 2 | 3 | 145 | 21 | 298 | 224 | 76 | 42 | 8 | 15 | 953 |
| DL3C | 18 | 16 | 84 | 2 | 2 | 145 | 21 | 284 | 214 | 73 | 39 | 8 | 14 | 920 |
| SH1A | 15 | 10 | 70 | 1 | 3 | 123 | 17 | 250 | 190 | 74 | 34 | 5 | 9 | 801 |
| SH1B | 18 | 13 | 71 | 2 | 3 | 125 | 17 | 276 | 207 | 75 | 34 | 5 | 9 | 855 |
| SH1C | 17 | 14 | 75 | 2 | 3 | 131 | 17 | 276 | 211 | 77 | 33 | 5 | 10 | 871 |
| SH2A | 22 | 15 | 86 | 1 | 4 | 146 | 18 | 282 | 237 | 79 | 43 | 4 | 11 | 948 |
| SH2B | 19 | 14 | 78 | 1 | 4 | 133 | 17 | 261 | 214 | 75 | 32 | 4 | 8 | 860 |
| SH2C | 22 | 16 | 90 | 1 | 3 | 150 | 17 | 285 | 238 | 79 | 44 | 3 | 11 | 959 |
| SZ1A | 30 | 25 | 116 | 2 | 4 | 204 | 24 | 358 | 328 | 119 | 63 | 13 | 22 | 1308 |
| SZ1B | 28 | 21 | 118 | 2 | 5 | 192 | 24 | 349 | 310 | 112 | 63 | 14 | 22 | 1260 |
| SZ1C | 31 | 25 | 122 | 2 | 5 | 209 | 23 | 361 | 328 | 119 | 65 | 13 | 22 | 1325 |
| SZ2A | 34 | 28 | 130 | 2 | 5 | 214 | 23 | 368 | 336 | 121 | 67 | 13 | 23 | 1364 |
| SZ2B | 32 | 28 | 124 | 2 | 5 | 216 | 26 | 370 | 343 | 116 | 70 | 14 | 24 | 1370 |
| SZ2C | 30 | 27 | 126 | 2 | 5 | 216 | 26 | 364 | 336 | 122 | 66 | 14 | 25 | 1359 |
| SZ3A | 31 | 26 | 121 | 2 | 6 | 210 | 26 | 376 | 338 | 120 | 66 | 13 | 23 | 1358 |
| SZ3B | 28 | 28 | 121 | 2 | 6 | 198 | 23 | 364 | 317 | 117 | 64 | 12 | 21 | 1301 |
| SZ3C | 28 | 27 | 122 | 2 | 4 | 206 | 25 | 365 | 323 | 120 | 63 | 14 | 21 | 1320 |
| WX1A | 19 | 15 | 76 | 2 | 4 | 138 | 19 | 269 | 212 | 77 | 41 | 5 | 9 | 886 |
| WX1B | 16 | 16 | 75 | 1 | 3 | 133 | 19 | 256 | 202 | 75 | 36 | 4 | 9 | 845 |
| WX1C | 20 | 16 | 71 | 2 | 3 | 128 | 17 | 263 | 213 | 81 | 41 | 4 | 11 | 870 |
| WX2A | 18 | 15 | 73 | 1 | 4 | 128 | 18 | 235 | 195 | 69 | 33 | 5 | 10 | 804 |
| WX2B | 19 | 14 | 77 | 1 | 4 | 132 | 18 | 263 | 205 | 79 | 38 | 6 | 11 | 867 |
| WX2C | 19 | 13 | 74 | 1 | 3 | 130 | 19 | 260 | 199 | 77 | 35 | 4 | 10 | 844 |
| ZZ1A | 11 | 13 | 64 | 1 | 4 | 108 | 15 | 219 | 170 | 59 | 26 | 3 | 5 | 698 |
| ZZ1B | 15 | 16 | 74 | 2 | 4 | 124 | 16 | 253 | 207 | 73 | 34 | 4 | 10 | 832 |
| ZZ1C | 14 | 12 | 67 | 2 | 4 | 103 | 15 | 239 | 184 | 68 | 29 | 4 | 9 | 750 |
| ZZ2A | 16 | 13 | 67 | 2 | 3 | 113 | 18 | 255 | 192 | 72 | 30 | 4 | 6 | 791 |
| ZZ2B | 12 | 10 | 61 | 1 | 2 | 105 | 16 | 240 | 170 | 64 | 27 | 3 | 7 | 718 |
| ZZ2C | 17 | 12 | 66 | 2 | 2 | 119 | 17 | 248 | 194 | 72 | 30 | 4 | 8 | 791 |
| ZZ3A | 15 | 10 | 65 | 1 | 3 | 95 | 15 | 231 | 178 | 63 | 27 | 3 | 6 | 712 |
| ZZ3B | 14 | 10 | 65 | 2 | 4 | 112 | 16 | 240 | 186 | 62 | 29 | 4 | 8 | 752 |
| ZZ3C | 12 | 10 | 69 | 2 | 4 | 111 | 17 | 244 | 181 | 63 | 29 | 4 | 9 | 755 |

The alphabets (DL, BJ, ZZ, CS, WX, SH, SZ) represent the cities and the number represents the number of the WWTPs. ‘A’, “B”, “C” following number mean three replicates in the same WWTP respectively.
