# Supplementary material for: The Composition and Spatial Patterns of Bacterial Virulence Factors and Antibiotic Resistance Genes in 19 Wastewater Treatment Plants
Source: PLoS One. 2016 Dec 1;11(12):e0167422. doi: 10.1371/journal.pone.0167422 (PMC5132249; doi:10.1371/journal.pone.0167422)
Supplement: S4 Table — The alphabets (DL, BJ, ZZ, CS, WX, SH, SZ) represent the cities and the number represents the number of the WWTPs. ‘A’, “B”, “C” following number mean three replicates in the same WWTP respectively. (DOCX) [file pone.0167422.s006.docx]

**S4 Table. Diversity indices from 57 samples**

| Samples | Shannon Index  (H) | Simpson Index  (D) | Pielou evenness  (J) |
| --- | --- | --- | --- |
| BJ1A | 6.55 | 699.73 | 0.9998 |
| BJ1B | 6.65 | 774.43 | 0.9998 |
| BJ1C | 6.64 | 764.28 | 0.9997 |
| BJ2A | 6.75 | 851.95 | 0.9997 |
| BJ2B | 6.71 | 816.98 | 0.9997 |
| BJ2C | 6.76 | 861.97 | 0.9997 |
| BJ3A | 6.58 | 722.84 | 0.9998 |
| BJ3B | 6.66 | 782.49 | 0.9998 |
| BJ3C | 6.64 | 764.16 | 0.9996 |
| BJ4A | 6.73 | 839.64 | 0.9997 |
| BJ4B | 6.72 | 829.76 | 0.9997 |
| BJ4C | 6.72 | 824.46 | 0.9997 |
| CS1A | 6.82 | 909.37 | 0.9996 |
| CS1B | 6.80 | 890.81 | 0.9996 |
| CS1C | 6.80 | 898.57 | 0.9996 |
| CS2A | 6.72 | 826.41 | 0.9996 |
| CS2B | 6.56 | 705.56 | 0.9996 |
| CS2C | 6.68 | 795.31 | 0.9996 |
| DL1A | 6.96 | 1044.75 | 0.9994 |
| DL1B | 6.97 | 1062.93 | 0.9995 |
| DL1C | 6.84 | 933.32 | 0.9996 |
| DL2A | 6.85 | 943.21 | 0.9996 |
| DL2B | 6.89 | 983.28 | 0.9995 |
| DL2C | 6.90 | 991.96 | 0.9995 |
| DL3A | 6.83 | 925.82 | 0.9995 |
| DL3B | 6.86 | 947.51 | 0.9995 |
| DL3C | 6.82 | 914.95 | 0.9995 |
| SH1A | 6.68 | 797.94 | 0.9996 |
| SH1B | 6.75 | 851.48 | 0.9996 |
| SH1C | 6.77 | 867.04 | 0.9996 |
| SH2A | 6.85 | 943.58 | 0.9996 |
| SH2B | 6.76 | 856.50 | 0.9996 |
| SH2C | 6.86 | 954.95 | 0.9996 |
| SZ1A | 7.17 | 1296.90 | 0.9994 |
| SZ1B | 7.14 | 1250.56 | 0.9994 |
| SZ1C | 7.19 | 1314.70 | 0.9994 |
| SZ2A | 7.21 | 1352.91 | 0.9993 |
| SZ2B | 7.22 | 1358.99 | 0.9993 |
| SZ2C | 7.21 | 1348.58 | 0.9994 |
| SZ3A | 7.21 | 1348.26 | 0.9994 |
| SZ3B | 7.17 | 1289.37 | 0.9993 |
| SZ3C | 7.18 | 1310.40 | 0.9994 |
| WX1A | 6.79 | 882.47 | 0.9996 |
| WX1B | 6.74 | 841.76 | 0.9996 |
| WX1C | 6.77 | 866.72 | 0.9996 |
| WX2A | 6.69 | 801.86 | 0.9997 |
| WX2B | 6.76 | 863.87 | 0.9997 |
| WX2C | 6.74 | 841.04 | 0.9997 |
| ZZ1A | 6.55 | 694.88 | 0.9997 |
| ZZ1B | 6.72 | 828.68 | 0.9996 |
| ZZ1C | 6.62 | 746.79 | 0.9996 |
| ZZ2A | 6.67 | 786.05 | 0.9995 |
| ZZ2B | 6.58 | 715.33 | 0.9996 |
| ZZ2C | 6.67 | 787.06 | 0.9995 |
| ZZ3A | 6.57 | 709.24 | 0.9996 |
| ZZ3B | 6.62 | 748.43 | 0.9995 |
| ZZ3C | 6.63 | 751.62 | 0.9996 |

The alphabets (DL, BJ, ZZ, CS, WX, SH, SZ) represent the cities and the number represents the number of the WWTPs. ‘A’, “B”, “C” following number mean three replicates in the same WWTP respectively.
